# Interleukin-6 is a key factor for immunoglobulin-like transcript-4-mediated immune injury in sepsis

**DOI:** 10.1186/s40560-018-0294-8

**Published:** 2018-04-10

**Authors:** De Wen Zhang, Jian He

**Affiliations:** Department of Emergency and Critical Care Medicine, Eastern Hepatobiliary Surgery Hospital, Second Military Medical University, No. 700 North Moyu Road, Shanghai, 201805 China

**Keywords:** Sepsis, Monocytes, Immunoglobulin-like transcript-4 (ILT4), Major histocompatibility complex class II molecules (MHC-II), Interleukin (IL)-6

## Abstract

**Background:**

ILT4^+^ monocytes seem to be associated with poor prognosis of sepsis in humans, but the exact mechanisms are unknown. This study aimed to examine the biological behaviors and effects of immunoglobulin-like transcript-4 (ILT4) levels on monocytes during sepsis and on the prognosis of sepsis.

**Methods:**

ILT4^+/+^ (WT) and ILT4-knockout (ILT4^−/−^) male BALB/c mice were used for sepsis modeling using cecal ligation puncture (CLP). Flow cytometry was used to measure the levels of ILT4 and major histocompatibility complex class II (MHC-II) on peripheral blood monocytes 24 h after CLP. ELISA was used to measure the serum levels of tumor necrosis factor-alpha (TNF-α), interleukin (IL)-1β, IL-6, and IL-12 at 0, 6, 12, and 24 h after CLP. Survival and prognosis were monitored over the course of 168 h.

**Results:**

ILT4 was highly expressed in peripheral blood monocytes of septic mice 24 h after CLP (1292.00 ± 143.70 vs. 193.50 ± 52.54, *p* < 0.05). MHC-II levels on peripheral blood monocytes in ILT4^−/−^ mice were significantly higher than those in WT mice (49.38 ± 5.66% vs. 24.25 ± 6.76%, *p* < 0.05). Serum IL-6 was significantly elevated 24 h after CLP (470.75 ± 88.03 vs. 54.25 ± 20.04, *p* < 0.05). The serum IL-6 levels were significantly lower in ILT4^−/−^ mice compared with those in WT mice after CLP (241.25 ± 45.10 vs. 470.75 ± 88.03, *p* < 0.05), but TNF-α, IL-1β, and IL-12 were not changed. The survival of ILT4^−/−^ mice was significantly better after CLP compared with that of WT mice.

**Conclusions:**

High levels of ILT4 on monocytes were observed in peripheral blood during sepsis and found to be associated with high serum IL-6 levels and low MHC-II levels on monocytes, possibly associated with higher mortality. ILT-4-IL-6-MHC-II could be a potential signaling pathway involved in sepsis.

## Background

Sepsis is a life-threatening condition caused by dysregulated host response to infection [1]. Ever since the concept of sepsis was proposed in 1991, the understanding of its pathogenesis and clinical patterns has made great progress. Mortality from sepsis remains high, and the management of severe sepsis is still a clinical challenge [2]. A previous study in China reported an incidence of sepsis in surgical intensive care units (ICU) of 8.68% and a mortality rate of 48.7% [3].

The pathogenesis of sepsis is very complex, involving derangements in infection, inflammation, immunity, and coagulation [4]. Studies have shown that the poor prognosis of sepsis is not entirely and directly caused by the pathogens or their toxins. Indeed, the immune responses of the host also play important roles in the progression of sepsis. The innate immune response is activated through pattern-recognition receptors (PRRs) and pathogen-pathogen interaction. These PRRs also react with some danger-associated molecular patterns (DMAPs), leading to excessive inflammatory response and immune system imbalance [5, 6]. Sepsis is a biphasic process showing first a hyper-inflammatory phase, followed by a hypo-inflammatory phase characterized by monocyte deactivation [7]. Sepsis after major surgery has been associated with defects in the production of monocyte cytokines [8]. The exact mechanisms of this deactivation are still poorly understood.

Immunoglobulin-like transcripts (ILTs), also known as leukocyte immunoglobulin-like receptors (LiLRs), monocyte/macrophage Ig-like receptor (MIRs), and CD85, are a family of genes encoded on the human chromosome 19q13.4 [9]. ILT4 is mainly expressed on monocytes/macrophages in the peripheral blood. It is a transmembrane protein with a long cytoplasmic tail containing two to four immunoreceptor tyrosine-based inhibitory motifs (ITIMs) to deliver inhibitory signals through protein tyrosine phosphatase (SHP) [10]. Upregulation of ILT4 in the serum of septic patients is directly correlated with the degree of organ dysfunction [11]. ILT4^+^ monocytes from septic patients display an alteration in the cytokine response to endotoxin stimulation [11]. Furthermore, ILT4 participates in the regulation of neutrophils in inflammatory disorders [12].

Therefore, we hypothesized that ILT4 is involved in the deregulation of the monocytes during sepsis. The aim of the present study was to determine the biological behaviors and effects of ILT4 levels on peripheral blood monocytes of septic mice.

## Methods

### Animals

Male 8-week-old wild type (WT) and ILT4-knockout (ILT4^−/−^) mice of the BALB/c background (weight, 25–30 g) were used in this study. The mouse homology of ILT4 is PIR-B [13]. To be discussed conveniently in this paper, the mice will be referred to as ILT4. WT BALB/c mice were purchased from Shanghai Laboratory Animal Center of Chinese Academy of Science (Shanghai, China). The ILT4^−/−^ mice were generated and bred by MultiSciences/Lianke Biotech Co., Ltd. (Zhejiang Province, China). The genotype of each mouse used in this study had been confirmed by PCR genotyping of tail DNA before the study.

### Sepsis modeling with CLP

Based on a previous study [14], sepsis modeling was conducted using cecal ligation puncture (CLP) in BALB/c mice. Animals were fasted for 12 h, followed by anesthesia with 10% chloral hydrate intraperitoneal injection (0.5 ml/100 g bodyweight) and abdominal midline incision (1.5 cm length) to ligate the cecum at the three-fourth point from its free end using no. 4 suture thread. A 21 gauge needle was used to perforate the midpoint of the ligated area along the longitudinal axis of the mesentery to extrude a small drop of intestinal content from each of the two puncture holes. The bowel was then put back in the abdominal cavity, which was sutured layer by layer. Animals received subcutaneous injection of 37 °C normal saline (5 mL/100 g bodyweight) after surgery and were housed in the animal facility with 12 h/12 h circadian rhythm and free access to water and food.

### Specimen collection

Blood samples were collected by heart puncture at 0, 6, 12, and 24 h after CLP and centrifuged at 2500 rpm for 20 min after sitting at room temperature for 20 min for serum collection. Serum samples were stored at − 20 °C. Whole blood samples collected at 24 h after CLP were stored in tubes containing EDTA anticoagulant for immediate flow cytometry.

### Flow cytometry analysis of ILT4 and MHC-II on monocytes

Red blood cells from 50 μL of EDTA-anticoagulated blood were lysed to prepare a cell suspension (1 × 10^8^ cells/ml) after washing, followed by fluorescent labeling for CD14 (1 μL of APC-CD14 monoclonal antibody, eBioscience, San Diego, CA, USA), ILT4 (2 μL of PE-ILT4 monoclonal antibody, eBioscience), and MHC-II (2 μL of FITC-MHC-II monoclonal antibody, eBioscience) separately and incubated at room temperate according to the manufacturer’s instructions. Then, levels of ILT4 and MHC-II in monocytes were quantified using a MACSQuant flow cytometry system (Miltenyi Biotec GmbH, Bergisch Gladbach, Germany).

### ELISA detection of serum tumor necrosis factor-alpha (TNF-α), interleukin (IL)-1β, IL-6, and IL-12

Fifty microliters of each serum sample was added into wells of ELISA plates. Buffer, biotinylated anti-mouse TNF-α, IL-1β, IL-6, and IL-12, horseradish peroxidase (HRP)-labeled streptavidin, and 3,3′,5,5′-tetramethylbenzidine (TMB) substrate were added according to the instructions included with the kit (Bender, MedSystems GmbH, Vienna, Austria). The plates were shaken and incubated at room temperature. After color development was complete, the stopping solution was added to terminate the reaction and optical density (OD) was immediately measured at 450 nm (using 650 nm as reference wavelength) using a Multiskan Ascent microplate reader (LabSystems Diagnostic Ltd., Helsinki, Finland) to calculate the concentrations using the standard curve (prepared using different dilutions of standard sample provided in the kit).

### Survival and prognosis

Twenty animals from each of the BALB/c WT and ILT4^−/−^ groups were evaluated. The number of surviving mice was recorded every 12 h after sepsis modeling by CLP, for a total of 168 h.

### Statistical analyses

SPSS 18.0 (IBM, Armonk, NY, USA) was used for statistical analysis. Continuous data were presented as mean ± standard deviation and analyzed using the Student *t* test and two-way ANOVA. Cox-Mantel log-rank and Breslow tests were used for statistical analysis of mouse survival. *p* < 0.05 was considered indicative of significant differences.

## Results

### Monocytes highly expressed ILT4 during sepsis

**Fig. 1:**
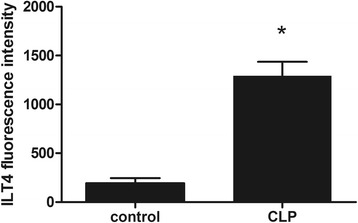
ILT4 fluorescence intensity in CD14^+^ monocytes 24 h after CLP. Note: *p < 0.05, significant difference between groups, *n* = 8 per group

ILT4 was highly expressed on peripheral blood monocytes of septic mice 24 h after CLP (1292.00 ± 143.70 vs. 193.50 ± 52.54, + 566%, *p* < 0.05, Fig. 1).

### ILT4 inhibited MHC-II levels on monocytes during sepsis

**Fig. 2:**
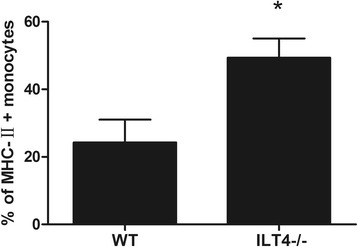
Percentage of monocytes expressing MHC-II 24 h after CLP. Note: *p < 0.05, significant difference between groups, *n* = 8 per group

MHC-II levels on peripheral blood monocytes in ILT4^−/−^ mice were significantly higher than those in WT mice (49.38 ± 5.66% vs. 24.25 ± 6.76%, + 103%, *p* < 0.05, Fig. 2).

### ILT4 regulated IL-6 but not TNF-α, IL-1β, or IL-12 levels during sepsis

**Fig. 3:**
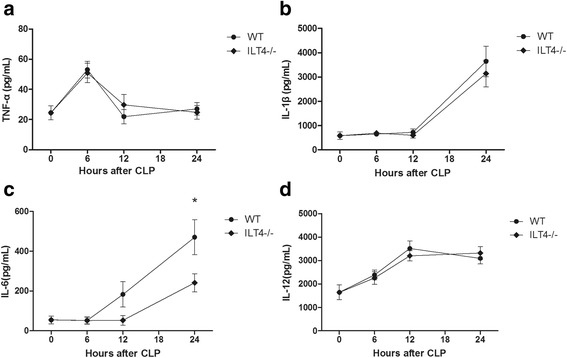
Serum TNF-α, IL-1β, IL-6, and IL-12 levels (pg/mL) at 0, 6, 12, and 24 h after CLP. **a** TNF-α, **b** IL-1β, **c** IL-6, and **d** IL-12. Note: *p < 0.05, significant difference between groups, *n* = 8 per group

Serum TNF-α showed a one-way trend peaking at 6 h after CLP (53.13 ± 5.49 vs. 24.50 ± 4.57, + 117%, *p* < 0.05) that reverted to preoperative levels 12 h after CLP in WT mice. ILT4 knockout showed no significant impact of CLP on TNF-α levels (50.88 ± 6.38 vs. 53.13 ± 5.49, − 4%, *p* > 0.05, Fig. 3a). Twenty-four hours after CLP, serum IL-1β was significantly elevated in the WT mice (3639.13 ± 627.20 vs. 581.75 ± 152.89, + 525%, *p* < 0.05) while serum IL-1β was not affected in ILT4^−/−^ mice (3144.63 ± 549.74 vs. 3639.13 ± 627.20, − 14%, *p* > 0.05, Fig. 3b). IL-6 was significantly elevated 24 h after CLP in the WT mice (470.75 ± 88.03 vs. 54.25 ± 20.04, + 772%, *p* < 0.05). The IL-6 levels were significantly lower in ILT4^−/−^ mice compared with those in WT mice after CLP (241.25 ± 45.10 vs. 470.75 ± 88.03, − 49%, *p* < 0.05, Fig. 3c). Serum IL-12 levels were significantly high 12 h after CLP in WT mice compared with controls (3508.99 ± 326.77 vs. 1641.57 ± 314.13, + 114%, *p* < 0.05) but was not significantly affected by ILT4 knockout (3198.74 ± 221.12 vs. 3508.99 ± 326.77, − 9%, *p* > 0.05, Fig. 3d).

### High levels of ILT4 increased the mortality of sepsis

**Fig. 4:**
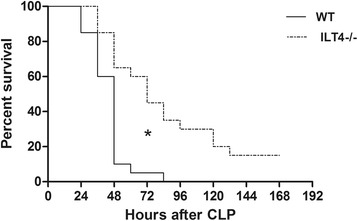
Kaplan-Meier survival curves of the different groups of mice showing relative survival in the different groups every 12 h, for a total 168 h. Note: *p < 0.05, significant difference between groups, *n* = 20 per group

The survival of ILT4^−/−^ mice after CLP was significantly higher than that of WT mice (*p* < 0.05, Fig. 4).

## Discussion

Sepsis is a complex pathophysiological process caused by the interaction between the host and pathogens. Monocytes are the main effector cells in innate immunity and antigen presentation and show diverse manifestations in infectious diseases according to their subtype and stimulation by lipopolysaccharide (LPS). In the past, immunophenotypes and biological behaviors of monocytes were roughly divided into two groups [15]. CD14^++^ CD16^−^ “classical monocytes” are the dominant subgroup under normal circumstances, accounting for 90–95% of the total monocytes in healthy humans. LPS stimulation leads to more active phagocytosis and causes the synthesis and secretion of IL-10 [16], which is anti-inflammatory. CD14^+^ CD16^+^ “non-classical monocytes” account for 5–10% of the total monocytes in healthy humans. LPS can induce the massive secretion of TNF-α by non-classical monocytes [16, 17]. The populations of these cell subgroups increases during infection and sepsis and are positively correlated with the poor prognosis of sepsis [18]. In the present study, the specific high ILT4 levels on peripheral blood monocytes during sepsis was associated with high serum IL-6 concentrations and low MHC-II levels on monocytes, leading to poor prognosis. Nevertheless, the specific properties of these cell populations have not been clarified yet, but Baffari et al. [11] showed that ILT4 is crucial to the tolerogenic function of monocytes. Indeed, high levels of ILT4 appear to be induced by soluble factors present in the serum of septic patients and directly correlates with the degree of organ dysfunction in human subjects [11]. ILT4^+^ monocytes from septic patients also display an alteration in the cytokine response to endotoxin stimulation characterized by reduced IL-12 production and increased IL-10 production [11]. Therefore, ILT4 probably plays important roles in sepsis.

In this study, ILT4 was highly expressed by peripheral blood monocytes of septic mice 24 h after CLP, while mortality of ILT4-knockout sepsis mice was significantly reduced. In view of this phenomenon, this study detected the relevant inflammatory mediators and antigen-presenting cells in order to identify the potential pathogenic mechanisms of ILT4. The results showed that serum IL-6 was significantly increased 24 h after CLP, and ILT4 knockout significantly suppressed this phenomenon, which was negatively correlated with survival. These findings were consistent with the clinical study by Gomez et al. [19]. An earlier study suggested that septic patients with serum IL-6 > 1000 pg/mL had 56% mortality, while patients with < 1000 pg/mL serum IL-6 had a mortality of only 40% [20].

Nevertheless, ILT4 had no significant impact on other inflammatory mediators, including TNF-α, IL-1β, and IL-12. ILT4 gene knockout demonstrated significant protective effects on septic mice at high serum levels of TNF-α, IL-1β, and IL-12, which was surprisingly different from our expectations. High levels of TNF-α [21] and IL-1β [22] in sepsis are generally considered to be correlated with high mortality, while IL-12, the primary effector of monocytes, can synergize IFN-γ to promote the inflammatory response, leading to poor prognosis of sepsis [23]. This could be explained by the existence of a numerous inflammatory mediators during sepsis, each playing multiple roles in a complex network and it is still unclear which are the ultimate key factors. This study showed that the effects of IL-6 were independent from the effects of TNF-α, IL-1β, and IL-12, but IL-6 was regulated by ILT4, which was associated with poor prognosis of sepsis. Further investigation of the regulation of IL-6 levels by ILT4 and its correlation with poor prognosis should be conducted in the future.

This study also showed that MHC-II levels on monocytes were significantly higher in the peripheral blood of ILT4^−/−^ mice than in WT mice. MHC-II mediates the transmission of extracellular signals. Following bacterial infection, macrophages phagocytose the bacteria and use MHC-II to supply T helper cells with bacterial fragments to initiate the immune response. In this way, MCH-II plays an essential role in the response to infection. Indeed, patients with < 30% HLA-DR (a member of the MHC-II family) in monocytes had 100% mortality, while patients with < 40% HLA-DR in monocytes had 80% mortality [24, 25]. High MHC-II levels on monocytes rather than the reduction of serum IL-6 was here found to be key to the survival of the ILT4^−/−^ mice. Nevertheless, the correlation between IL-6 and MHC-II has been reported [26].

The present study is not without limitations. The experiments were performed in the CLP animal model of sepsis, which is of course not perfect and could not completely mimic what is observed in humans.

## Conclusions

In conclusion, this study showed that high levels of ILT4 in monocytes during sepsis were associated with high serum IL-6 levels and low MHC-II levels on monocytes, resulting in higher mortality of sepsis. This study provides a new focus and further understanding of the pathology of sepsis. Nevertheless, further in-depth studies of the interplay among cytokines and molecular pathways are necessary.

## Abbreviations

CLP: Cecal ligation puncture
DMAPs: Danger-associated molecular patterns
IL: Interleukin
ILT4: Immunoglobulin-like transcript-4
ITIMs: Immunoreceptor tyrosine-based inhibitory motifs
LPS: Lipopolysaccharide
MHC-II: Major histocompatibility complex class II molecules
PRRs: Pattern-recognition receptors
TNF-α: Tumor necrosis factor-alpha
